# Variation in leaf morphological, stomatal, and anatomical traits and their relationships in temperate and subtropical forests

**DOI:** 10.1038/s41598-019-42335-2

**Published:** 2019-04-09

**Authors:** Congcong Liu, Ying Li, Li Xu, Zhi Chen, Nianpeng He

**Affiliations:** 1 Key Laboratory of Ecosystem Network Observation and Modeling, Institute of Geographic Sciences and Natural Resources Research, Chinese Academy of Sciences, Beijing, 100101 China; 2 University of Chinese Academy of Sciences, Beijing, 100049 China; 3 The Key Laboratory for Forest Resources& Ecosystem Processes of Beijing, Beijing Forestry University, Beijing, China; 4 Institute of Grassland Science, Northeast Normal University and Key Laboratory of Vegetation Ecology, Ministry of Education, Changchun, 130024 China

## Abstract

Leaf functional traits have attracted the attention of ecologists for several decades, but few studies have systematically assessed leaf morphological traits (termed “economic traits”), stomatal (termed “hydraulic”), and anatomical traits of entire forest communities, thus it is unclear whether their relationships are consistent among trees, shrubs, and herbs, and which anatomical traits should be assigned to economical or hydraulic traits. In this study, we collected leaf samples of 106 plant species in temperate forests and 164 plant species in subtropical forests and determined nine key functional traits. We found that functional traits differed between temperate and subtropical forests. Leaf traits also differed between different plant functional groups, irrespective of forest type; dry matter content, stomatal density, and cell tense ratio followed the order trees > shrubs > herbs, whereas specific leaf area and sponginess ratio showed the opposite pattern. The correlations of leaf traits were not consistent among trees, shrubs, and herbs, which may reflect different adaptive strategies. Principal component analysis indicated that leaf economics and hydraulic traits were uncoupled in temperate and subtropical forests, and correlations of anatomical traits and economic and hydraulic traits were weak, indicating anatomical traits should be emphasized in future studies.

## Introduction

Plant functional traits are commonly used as robust indicators of factors affecting the distribution of species, and they can be used to predict the responses of ecosystem structures and functions to global climate change^1^. Leaf functional traits are one of the most important group of plant functional traits, and due to their conspicuousness and ease of measurement in the field in numerous species these traits have been well-studied previously^2^. Recently, Wang, *et al*.^3^ tested the relationships of leaf, stem, and root traits of shrubs and concluded that leaf traits are a robust proxy for traits of the entire plant. Therefore, strategies of adaption to the environment may largely be reflected in leaf functional traits^4^.

Studies on leaf functional traits have contributed to the understanding of plant adaptation to changing environments. Previous studies indicated that specific leaf area increased with decreasing light exposure^5,6^, leaf thickness increased due to intense irradiance^7^, the thickness of palisade tissue decreased and that of spongy tissue increased under weak lighting conditions^8^, and higher maximum stomatal conductance may benefit species under low CO_2_ conditions and high irradiance or nutrient supply^9^. Cornwell, *et al*.^10^ and Cornwell and Ackerly^11^ used leaf functional traits to predict how plant species co-exist within a community and to elucidate the drivers of community assembly. However, all of these studies were conducted on a small array of plant species or focused on only one or few leaf functional traits, and few studies combined leaf morphological (referred to as “economical”), stomatal (referred to as “hydraulic”), and anatomical traits of an entire forest community.

Relationships between leaf functional traits have been examined in different leaf types, phylogenetic groups, biomes, and even on a global scale. Reich, *et al*.^12^ found that relationships between pairs of leaf functional traits exhibited similar trends in different ecosystems, suggesting predictable relationships between leaf functional traits. On a global scale, Wright, *et al*.^13^ reported that relationships between leaf traits were robust, and that climate had a surprisingly modest effect on trait correlations. However, La Riva, *et al*.^14^ found that the correlation of leaf mass per area and nutrient concentrations in 98 Mediterranean woody species was determined by phylogeny, habitat, and leaf habit. Franks, *et al*.^15^ reported that the correlation of stomatal size and leaf nitrogen concentration in *Eucalyptus globulus* differed between high and low rainfall conditions. As mentioned above, relationships between leaf functional traits tend to be more robust on a large geographic scale. Regarding trees, shrubs, and herbs in different environments, particularly under different lighting conditions, we hypothesized that relationships between leaf traits are not consistently stable in trees, shrubs, and herbs in forest communities.

Ever since a “leaf economic spectrum” was suggested^13^, studies on leaf functional traits have been taken to a new level. It has been debated whether leaf economic and hydraulic traits consistently co-vary or whether independent variation may occur, and Li, *et al*.^16^ found that leaf economic and hydraulic traits were statistically uncoupled in five tropical/subtropical forests, based on 85 woody species. However, Yin, *et al*.^17^ examined 47 woody species on the Chinese Loess Plateau and found that leaf economic and hydraulic traits were highly correlated. These opposing conclusions further obscured the relationships between these two categories of traits, and both of the studies mentioned included only woody species and no herbs. Moreover, it is unclear whether anatomical traits should be considered economic or the hydraulic traits^17^.

Here, we investigated nine key leaf functional traits, including leaf morphological or economic traits (dry matter content [DMC], specific leaf area [SLA], and leaf thickness [LT]), leaf stomatal or hydraulic traits (stomatal density [SD], stomatal pore length [SL], and stomatal pore index [SPI]), and anatomical traits (cell tense ratio [CTR], spongy tissue ratio [SR], and abaxial cell thickness [AB]) of 270 plant species in temperate and subtropical forests (Table 1), comprising 95 families and 216 genera (Supplementary Table S1). The main objectives of this study were 1) to quantify the nine leaf traits at the levels of species, plant functional groups and communities, 2) to test whether correlations of leaf functional traits were stable in trees, shrubs, and herbs in specific forest types, 3) to explore whether leaf economic and hydraulic traits were uncoupled in temperate and subtropical forests, and 4) to test correlations of leaf anatomical traits and the two other trait categories. Trait-based modelling has emerged to be one of the most promising approaches to simulate ecological processes, and our study may provide detailed input parameters for ecosystem modeling. In order to derive more general conclusions, it is insufficient to examine only one type of forest, thus subtropical and temperate forests were selected as they are the most typical forest types in China, and a comparative investigation may elucidate whether the above-mentioned hypotheses depend on the forest type.

**Table 1:** The nine measured traits and their categorization.

Traits	Abbr.	Unit	Group	Strategy
Specific leaf area	SLA	mm^2^ mg^−1^	Morphology	Resource capture and defense
Dry matter content	DMC	g kg^−1^	Morphology	Resource capture and defense
Leaf thickness	LT	mm	Morphology	Resource capture and defense
Stomatal pore length	SL	µm	Stomata	Gas exchange, *e*.*g*. CO_2_ and H_2_O
Stomatal density	SD	pores mm^−2^	Stomata	Gas exchange, *e*.*g*. CO_2_ and H_2_O
Stomatal pore index	SPI	%	Stomata	Gas exchange, *e*.*g*. CO_2_ and H_2_O
Cell tense ratio	CTR	%	Anatomy	Physiological process, structure
Spongy tissue ratio	SR	%	Anatomy	Physiological process, structure
Abaxial cell thickness	AB	µm	Anatomy	Structure and defense

## Results

### Differences in leaf morphological traits

**Figure 1:**
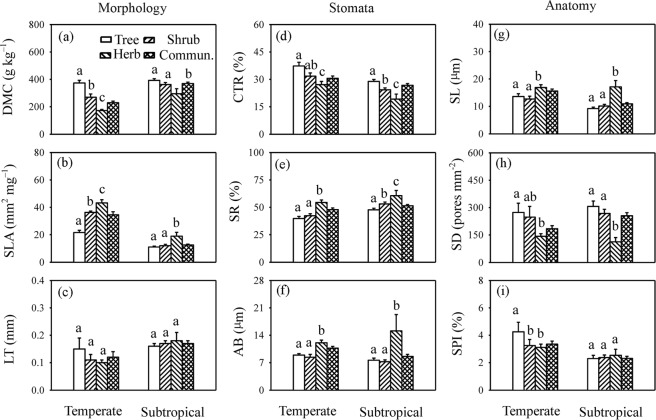
Differences in leaf traits between functional groups in temperate and subtropical forests. DMC, dry matter content; SLA, specific leaf area; LT, leaf thickness; SL, stomatal pore length; SD, stomatal density; SPI, stomatal area index; CTR, cell tense ratio; SR, spongy tissue ratio; AB, abaxial cell thickness. Data are shown as average ± SE. The same letters indicate no significant difference at the 0.05 level.

DMC ranged from 44.46 to 687.50 g kg^−1^ with an average of 229.25 g kg^−1^ in temperate forests (Figs 1 and S1), and in subtropical forests, it ranged from 82.27 to 896.55 g kg^−1^ with an average of 369.59 g kg^−1^ (Supplementary Fig. S1). The mean SLA was 34.52 mm^2^ mg^−1^ and 12.59 mm^2^ mg^−1^ in temperate and subtropical forests, respectively. The average LT was lower in temperate forests (0.12 mm) than in subtropical forests (0.17 mm). Regarding functional plant groups, DMC was the highest in trees, followed by shrubs and herbs, and the opposite pattern was found regarding SLA, irrespective of the forest type, whereas LT did not differ significantly between plant functional groups (Fig. 1a–c).

### Changes in leaf stomatal traits

Average SL in temperate forests was approximately 15.61 µm (Fig. 1), ranging from 4.78 to 47.32 µm (Supplementary Fig. S2); in subtropical forests, average SL was 10.91 µm, ranging from 2.79 to 43.40 µm. SD ranged from 14.88 to 961.31 pores mm^−2^ with a mean of 184.35 pores mm^−2^ in temperate forests (Supplementary Fig. S3), and SD of plants in subtropical forests ranged from 11.16 to 1403.27 pores mm^−2^ with an average of 255.12 pores mm^−2^. The average SPI was 3.35% and 2.31% in temperate and subtropical forests, respectively. SL and SPI were higher in temperate forests than in subtropical forests, whereas SD was higher in subtropical forests. Furthermore, SL was the highest in herbs, followed by shrubs and trees, and the opposite pattern was observed regarding SD (Fig. 1).

### Differences in leaf anatomical traits

CTR in plants in temperate forests ranged from 8.47 to 56.49%, with an average of 30.56% (Figs 1; S3), which by far exceeded that in plants in subtropical forests (average of 26.75%). Average SR was 47.89% and 51.37% in temperate and subtropical forests, respectively. AB in temperate forests ranged from 4.37 to 24.15 µm, with an average of 10.70 µm, and was significantly higher than in subtropical forests, ranging from 2.14 to 47.77 µm with an average of 8.60 µm. Regarding plant functional groups, the order of CTR was trees > shrubs > herbs, that of SR was trees < shrubs < herbs, and AB was highest in herbs, irrespective of the forest type (Fig. 1).

### Correlation of leaf functional traits and principal component analysis results in temperate and subtropical forests

**Figure 2:**
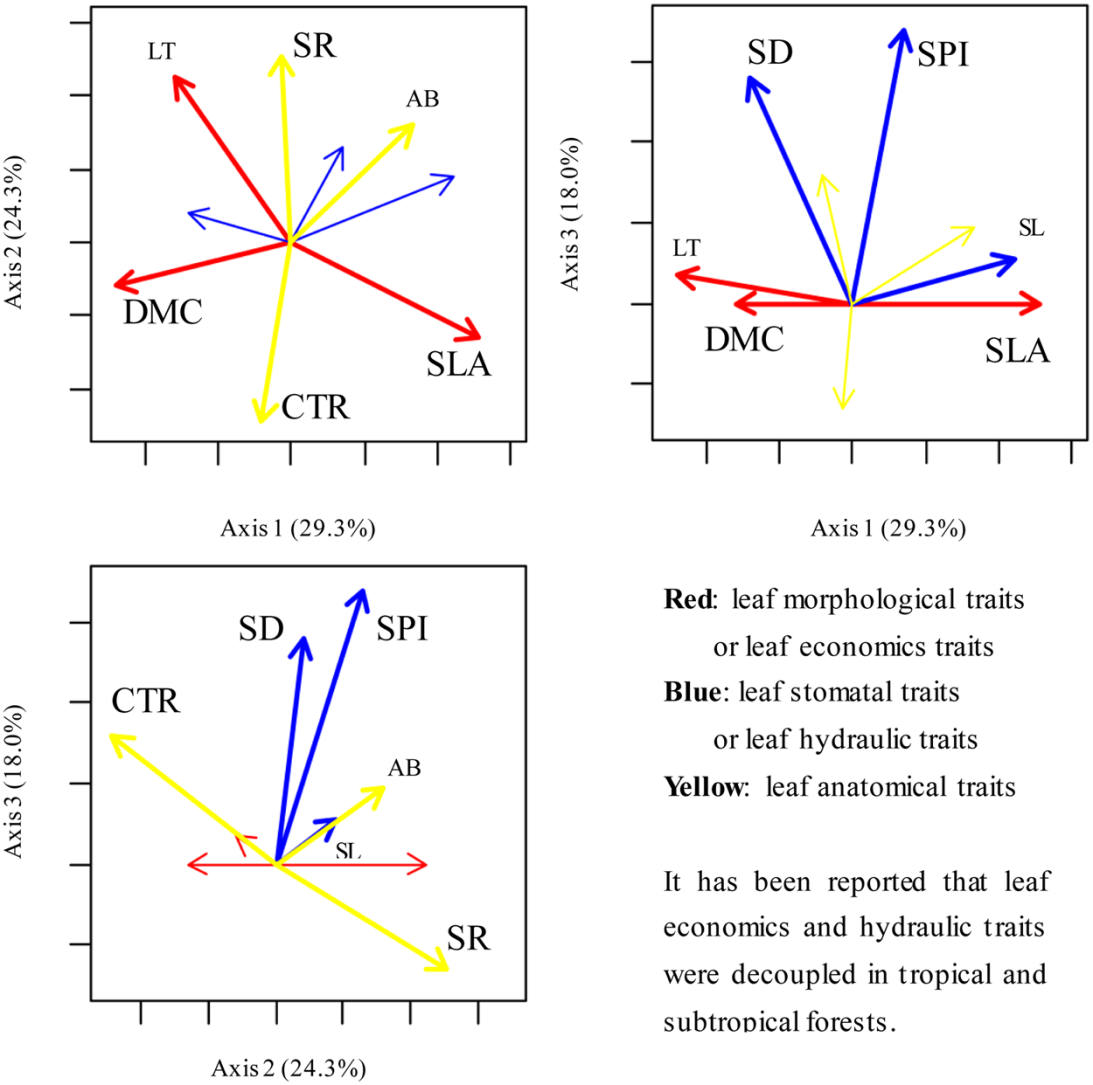
Principal component analyses. DMC, dry matter content; SLA, specific leaf area; LT, leaf thickness; SL, stomatal pore length; SD, stomatal density; SPI, stomatal area index; CTR, cell tense ratio; SR, spongy tissue ratio; AB, abaxial cell thickness.

Weak but significant correlations were observed among leaf economic, hydraulic, and anatomical traits (Table 2). The relationships between leaf traits were not always observed in trees, shrubs, and herbs, and some relationships only existed in specific plant functional groups and in one of the two forest types (Supplementary Figs S4–11. Principal component analysis (PCA) was employed to test the association of leaf economic, hydraulic, and anatomical traits (Fig. 2). PCA axis 1 explained 29.3% of the total variation and showed strong loadings from leaf morphological traits. PCA axis 2 explained 24.3% of the total variation and showed strong loadings from leaf anatomical traits. PCA axis 3 explained 18.0% of the total variation and showed strong loadings from leaf stomatal traits (Table 3 and Fig. 2).

**Table 2:** Pearson’s correlation of leaf traits.

	logSLA	logDMC	logLT	logSL	logSD	logSPI	logCTR	logSR	logAB
logSLA^†^		186^‡^	182	188	188	188	161	173	173
logDMC	−0.681**		193	248	248	248	194	212	214
logLT	−0.650**	0.174*		202	202	202	173	187	187
logSL	0.349**	−0.489**	−0.031		270	270	208	229	231
logSD	−0.219**	0.310**	0.067	−0.547**		270	208	229	231
logSPI	0.123	−0.196**	0.037	0.499**	0.452**		208	229	231
logCTR	0.065	0.251**	−0.267**	−0.200**	0.222**	0.026		207	206
logSR	−0.149	−0.096	0.233**	0.180**	−0.168*	0.015	−0.531**		227
logAB	0.237**	−0.488**	−0.006	0.469**	−0.255**	0.215**	−0.371**	0.142*	

^†^DMC, dry matter content; SLA, specific leaf area; LT, leaf thickness; SL, stomatal pore length; SD, stomatal density; SPI, stomatal pore index; CTR, cell tense ratio; SR, spongy tissue ratio; AB, abaxial cell thickness.

^‡^The numbers of samples are shown in the upper right section of the matrix. The correlation coefficients (r) are shown in the lower left section of the matrix. *P < 0.05; **P < 0.01.

**Table 3:** Loading scores of nine leaf functional traits in the principal component analyses (PCA) in temperate and subtropical forests.

Trait	Group	PC 1	PC 2	PC 3
SLA	Morphology	**0**.**51**	−0.26	0.00
DMC	Morphology	**−0**.**48**	−0.12	0.07
LT	Morphology	−0.32	0.45	0.00
SL	Stomata	0.44	0.18	0.11
SD	Stomata	−0.28	0.08	**0**.**56**
SPI	Stomata	0.14	0.26	**0**.**68**
CTR	Anatomy	−0.08	**−0**.**49**	0.32
SR	Anatomy	−0.03	**0**.**51**	−0.26
AB	Anatomy	0.33	0.32	0.19
% of variance		29.3	24.3	18.0

DMC, dry matter content; SLA, specific leaf area; LT, leaf thickness; SL, stomatal pore length; SD, stomatal density; SPI, stomatal pore index; CTR, cell tense ratio; SR, spongy tissue ratio; AB, abaxial cell thickness.

## Discussion

Economic, hydraulic, and anatomical leaf traits varied greatly between temperate and subtropical forests. Regarding economic traits, plants in subtropical forests had higher DMC and LT and lower SLA, compared with plants in temperate forests. A high DMC indicates little intercellular space and high mesophyll resistance to gas diffusion^18^; therefore, diffusion resistance in subtropical forest plants with a high DMC may be increased to decrease leaf transpiration. Higher LT can increase photosynthetic capacity under strong irradiance by increasing the nitrogen content and the volume of the photosynthetic machinery per unit leaf area, and thick leaves can prevent sunlight damage^19^, which may explain the higher LT in plants in subtropical forests. Lower SLA indicates higher construction cost per unit area, and SLA can reflect the potential of leaves to capture light, therefore higher SLA in plants in temperate forests may be an adaption to low light intensity.

Regarding hydraulic traits, subtropical forests had higher SD and SPI and smaller SL than plants in temperate forests. Smaller stomata have a higher surface area to volume ratio, so that they can respond quickly to environmental changes by opening and closing rapidly^20,21^; higher SD can help reduce CO_2_ diffusion resistance caused by a large mesophyll surface under strong irradiance^22^. This may explain small and dense stomata in plants growing in subtropical forests. Furthermore, the rates of cell division are severely constrained by low temperatures, which is reflected in larger and fewer stomata in plants in temperate forests^23^. In line with the results of Sack, *et al*.^24^, the SPI correlated with the maximum stomatal conductance. CO_2_ diffusion is reduced at low temperatures, therefore reduced stomatal conductance likely led to a larger SPI in temperate forests. Regarding leaf anatomical traits, temperate forest had thicker AB and larger CTR than those of subtropical forest. Thicker AB can reduce reflection of scattered light^25^ and larger CTR can enhance the cold resistance of plants^26^; therefore, temperate forest had a higher AB and larger CTR, which is likely an adaption to the cold climate and low light intensity.

The differences in leaf functional traits regarding morphology, stomata, and anatomy were significant between plant functional groups in temperate and subtropical forests. Regarding morphological traits, DMC produced the order trees > shrubs > herbs in both forest types. Higher DMC increases moisture diffusion resistance in leaves, and DMC is an indicator of leaf water content. Hydraulic effects are typically stronger in larger plants^27^, and DMC was indeed highest in trees, followed by shrubs and herbs. SLA showed the opposite pattern, which has previously been observed other in forest types^28–33^, and shaded leaves may increase the efficiency of light capture by increasing SLA^34^. This may be a result of the adaptation to declining light intensity form trees to shrubs to herbs. Regarding leaf stomatal traits, SL was highest in herbs, and SD showed the opposite pattern in both forest types, which was in line with the results of Wang, *et al*.^35^ and Liu, *et al*.^36^, probably because large stomata are critical for herbs to optimize light capture in light-limited environments^20^, and a higher SD increases the ability to regulate leaf transpiration^22^. Regarding leaf anatomical traits, CTR showed the order trees > shrubs > herbs, and SR produced the opposite pattern. Previous studies have shown that CTR decreased and SR increased with a decrease in light intensity^8,37^. Furthermore, photosynthesis occurs predominantly in fully developed palisade tissue^38^ and spongy tissue reduces the loss of photons and thus improve photosynthetic efficiency in low-light conditions^39^, which supports our observations of the highest CTR in trees and lowest CTR in herbs. Bone, *et al*.^40^ suggested that higher AB is an adaptation to low-light conditions as it can reduce the reflection of scattered light and increase irradiation intensity within the leaf, which indicates that plants with higher AB may be better adapted to light-limited environments.

Significant correlations of leaf functional traits were not entirely consistently in trees, shrubs, and herbs, and most correlations of leaf functional traits only occurred in specific plant functional groups; however, the correlation of SL and SD was observed in all plant functional groups, which may be explained by physical and energetic constraints^15,20^. Trait correlations may vary as a response to adaption to changing environments, and in a stable environment, multiple combinations of traits that are costly would not be required^17^. For instance, SLA was negatively correlated with DMC and LT, which indicated water retention ability because higher SLA may increase water loss but higher DMC and LT may increase moisture diffusion resistance and distance; however, these negative correlations were not observed in herbs in temperate forests, possibly due to low water loss in low-temperature and light-limited environments. Climate can shape and shift functional biodiversity in forests, and our results indicate that trait correlations may differ between environments, which may be an aspect to consider regarding global climate change.

Correlations of leaf economic and hydraulic traits were very weak, which was consistent with the results of Li, *et al*.^16^, but contradicted those of Yin, *et al*.^17^, which may be because water availability was not a limiting factor for plant growth at our study sites. Interestingly, we found that also the correlations of anatomical traits with economic and hydraulic traits were weak. Leaf anatomical traits and hydraulic traits were also uncoupled on the arid Loess Plateau (Supplementary Table S2), and numerous studies found that leaf anatomical traits closely correlated with temperature^41,42^, therefore temperature may be another factor to consider regarding anatomical adaptation strategies and resource usage by plants. As Li, *et al*.^16^ suggested, multi-dimensional adaptation strategies would be expected as an adaption to multifactorial changes in the environment.

## Conclusion

There are few systematic studies on the functional traits on entire forest communities. This is the first study to investigate leaf functional traits regarding morphology, stomata, and anatomy of an entire forest community. Our study demonstrated that trait correlations are not consistent, and leaf anatomical traits were uncoupled from leaf economic and hydraulic traits. Anatomical traits and trait co-variation should be further investigated in the future.

## Material and Methods

### Study site

The experiments were conducted in temperate and subtropical forests, as these account for 88.3% of the forest area of China. To avoid the effect of anthropogenic disturbance, sampling plots were established within well-protected national nature reserves in China where the vegetation was relatively homogenous and representative of the respective forest type^43^:

#### Temperate forests

Temperate forest plots were established in Changbai Mountain Natural Reserve (42°24′N, 128°05′E, 758 m.a.s.l.), China. The mean annual temperature was approximately 3.6 °C, and mean annual precipitation was 691 mm^44^. The soil was categorized as dark brown forest soil. The vegetation was typical of temperate forests with dominant tree species such as *Pinus koraiensis*, *Tilia amurensis*, and *Quercus mongolica*, among others, dominant shrubs such as *Corylus mandshurica*, *Acanthopanax senticosus*, and *Ribes mandshuricum*, and dominant herbs such as *Brachybotrys paridiformis* and *Phryma leptostachya*^45,46^.

#### Subtropical forests

Subtropical forest plots were established in the Dinghu Mountain Nature Reserve (N, 23°17′N, 112°54′E, 240 m.a.s.l.), China. The mean annual temperature was approximately 20.9 °C, and mean annual precipitation was 1955 mm^44^. The soil was characterized as a latosol. The vegetation type was considered a southern subtropical evergreen broad-leaved forest, with dominant trees *Castanopsis chinensis*, *Schima superba*, and *Cryptocarya chinensis*, dominant shrubs *Psychotria rubra* and *Ardisia punctata*, and dominant herbs *Chloranthus spicatus* and *Alpinia japonica*^46^.

### Sample collection

Samples were collected from July to August 2013. First, we established four plots (30 × 40 m) in each forest type. Geographic information (latitude, longitude, and altitude), plant species composition, and ecosystem structure were recorded for each plot. The number, height, diameter at breast height (≥2 cm) of all trees, basal stem diameter of shrubs, and coverage of herbs (and other data) were recorded. Leaf samples of trees, shrubs, and herbs were collected in and around the plots. Briefly, four healthy trees of each species were selected for collecting leaves from the middle and the canopy using long-handle shears or by climbing. From each plant species in each plot, 20 healthy mature leaves were collected from four individuals, representing one replicate. Leaf samples were placed in sealable plastic bags and immediately stored on ice in a cooling box. Measurements or pre-treatments were completed as soon as possible after collection (within 4–8 h).

In total, 106 plant species of temperate forests and 164 plant species of subtropical forests were collected, comprising 95 families and 216 genera (Table S1).

### Measurement of leaf traits

Leaf traits were assessed regarding morphology, stomata, and anatomy (Supplementary Fig. S12); units of measurement are shown in Table 1.

#### Morphological traits

After sampling, LT was measured using a Vernier caliper at an accuracy of 0.02 mm. Leaf area (LA) was measured in 16 leaves (four groups) using a scanner (Cano Scan LIDE 100, Japan) and Photoshop CS software (Adobe, USA). Leaf fresh weight (LFW) was measured using an electronic balance at an accuracy of 0.0001 g; the leaves were subsequently dried to constant weight in an oven^44^ to measure leaf dry weight (LDW). DMC (g kg^−1^) and SLA (mm^2^ mg^−1^) were calculated according to the following equations:

1 $${\rm{DMC}}=\frac{{\rm{LDW}}}{{\rm{LFW}}}\times 1000$$

2 $${\rm{SLA}}=\frac{{\rm{LA}}}{{\rm{LDW}}}$$

#### Stomatal traits

After field sampling, eight to ten clean leaves were cut into small pieces (1.0 × 0.5 cm) along the main vein. The pieces were then placed in a formalin acetic alcohol solution (50%; alcohol:formalin:glacial acetic acid:glycerin = 90:5:5:5) as soon as possible (3–6 h after collection) for analyzing leaf stomatal and anatomical traits.

We first examined the pre-treatment leaf samples in the laboratory after air-drying and scraping off surface hair using a razor blade. Stomatal traits were observed by scanning electron microscopy (Hitachi SN-3400, Hitachi, Tokyo, Japan). Three replicates of each species were examined, and two photographs of each replicate were taken in different positions. In each photograph, five stomata were randomly selected to measure SL using an electronic image analysis equipment (MIPS software, Optical Instrument Co., Ltd., Chongqing, China) and the number of stomata (N) in each photo was recorded^36^. SD and SPI were calculated as follows:

3 $${\rm{SD}}=\frac{{\rm{N}}}{1.12\times 1{0}^{-2}}$$

4 $${\rm{SPI}}={\rm{SD}}\times {{\rm{SL}}}^{2}\times 1{0}^{-4}$$

where 1.12 × 10^−2^ is the area of observed photo (mm^2^).

#### Anatomical traits

Samples fixed in a formalin acetic alcohol solution were serially dehydrated in ethanol (50–100%) and were then infiltrated using warm paraffin (56–58 °C). Leaf samples of 8–12 µm in size were produced using a rotary microtome (Leica RM 2255, Leica Instruments, Nussloch, Germany). The slides were stained using safranin and fast green (1% aqueous safranin and 0.5% fast green in 95% ethanol). Then, the sections were photographed to measure LT (in µm), palisade thickness (PT; in µm), spongy tissue thickness (ST; in µm), and AB (in µm) producing five measurements per photograph^41^. CTR and SR were calculated as follows:

5 $${\rm{CTR}}=\frac{{\rm{PT}}}{{\rm{LT}}}\times 100 \% $$

6 $${\rm{SR}}=\frac{{\rm{ST}}}{{\rm{LT}}}\times 100 \% $$

### Data analyses

The respective average of all leaf traits was calculated at the species level based on all replicates. The plant species were divided into three functional groups, namely trees, shrubs, and herbs. Then, leaf traits at the plant functional group and community level were calculated as the pooled mean of leaf traits per plant functional groups and community.

To compare the differences in leaf traits among different plant functional groups between forest types, a one-way analysis of variance with a least-significant-difference test was performed. A t-test for independent samples was used to test differences in leaf traits between forest types. Correlations of leaf traits were tested using Pearson’s correlation after log_10_-transformation of the data to meet the assumption of normal distribution. To test whether the relationships between leaf traits were consistent among trees, shrubs, and herbs, linear and non-linear regressions were fitted, and models with a lower Akaike’s information criterion values were chosen as the best-fitting models. Multivariate associations of leaf traits were analyzed with a PCA using R software (version 2.15.2, R Development Core Team 2012). Data analyses and visualization were performed using SPSS 13.0 (SPSS Inc., Chicago, IL, USA, 2004) and SigmaPlot 10.0 software (Systat, USA). Statistical significance is reported at *P* < 0.05.

## Supplementary information

SREP-18-32721-Supplementary

SREP-18-32721-dataset

## Data Availability

The datasets generated and analyzed in the current study are included in its Supplementary Information files.
