# Supplementary material for: Variation in leaf morphological, stomatal, and anatomical traits and their relationships in temperate and subtropical forests
Source: Sci Rep. 2019 Apr 9;9:5803. doi: 10.1038/s41598-019-42335-2 (PMC6456615; doi:10.1038/s41598-019-42335-2)
Supplement: Supplementary file 1 — SREP-18-32721-Supplementary [file 41598_2019_42335_MOESM1_ESM.pdf]

Variation in leaf morphological, stomatal, and anatomical traits and their relationships in temperate and subtropical forests

Congcong Liu [1,2*], Ying Li[3], Li Xu [1*], Zhi Chen[1], Nianpeng He[1,2,4]

[1] Key Laboratory of Ecosystem Network Observation and Modeling, Institute of Geographic Sciences and Natural Resources Research, Chinese Academy of Sciences, Beijing 100101, China;

[2] University of Chinese Academy of Sciences, Beijing 100049, China

[3]The Key Laboratory for Forest Resources& Ecosystem Processes of Beijing, Beijing Forestry University, Beijing, China

[4] Institute of Grassland Science, Northeast Normal University and Key Laboratory of Vegetation Ecology, Ministry of Education, Changchun 130024, China

*Correspondence and requests for materials should be addressed to Congcong Liu and Li Xu (1259803401@qq.com or xuli@igsnrr.ac.cn).

Tel.: +86-10-64889263;

Fax: +86-10-64889399.

Table S1Taxonomic information of the species sampled

Family	Genus
Aceraceae (3)	Acer (3)
Actinidiaceae (2)	Saurauia (1) Actinidia (1)
Adiantaceae (1)	Adiantum (1)
Anacardiaceae (2)	Dracontomelon (1) Rhus (1)
Annonaceae (1)	Fissistigma (1)
Aquifoliaceae (4)	Ilex (4)
Araceae (3)	Pinellia (1) Alocasia (1) Aglaonema (1)
Araliaceae (3)	Eleutherococcus (1) Aralia (1) Schefflera (1)
Aspidiaceae (1)	Hemigramma (1)
Athyriaceae (2)	Lunathyrium (1) Athyrium (1)
Begoniaceae (1)	Begonia (1)
Berberidaceae (2)	Berberis (1) Caulophyllum (1)
Betulaceae (1)	Corylus (1)
Blechnaceae (1)	Blechnum (1)
Boraginaceae (1)	Brachybotrys (1)
Brassicaceae (1)	Cardamine (1)
Burseraceae (2)	Canarium (2)
Caesalpiniaceae (3)	Erythrophleum (1) Caesalpinia (1) Bauhinia (1)
Caprifoliaceae (2)	Lonicera (1) Sambucus (1)
Caryophyllaceae (1)	Lychnis (1)
Celastraceae (2)	Euonymus (2)
Chloranthaceae (2)	Sarcandra (1) Chloranthus (1)
Compositae (12)	Artemisia (2) Saussurea (3) Prenanthes (1) Achillea (1) Adenocaulon (1) Arctium (1) Inula (1) Parasenecio (1) Sphagneticola (1)
Convolvulaceae (2)	Calystegia (1) Erycibe (1)
Cyatheaceae (1)	Alsophila (1)
Cycadaceae (1)	Cycas (1)
Cyperaceae (3)	Carex (2) Scleria (1)
Dicksoniaceae (1)	Cibotium (1)
Dilleniaceae (1)	Tetracera (1)
Dioscoreaceae (2)	Dioscorea (2)
Dryopteridaceae (1)	Dryopteris (1)
Ebenaceae (2)	Diospyros (2)
Elaeocarpaceae (1)	Elaeocarpus (1)
Equisetaceae (1)	Equisetum (1)
Ericaceae (4)	Ledum (1) Craibiodendron (1) Rhododendron (2)
Euphorbiaceae (10)	Euphorbia (1) Glochidion (1) Mallotus (1) Bridelia (1) Macaranga (1) Breynia (1) Alchornea (1) Croton (1) Antidesma (1) Aporosa (1)
Fabaceae (5)	Mucuna (1) Ormosia (1) Dalbergia (1) Callerya (1) Bowringia (1)

Fagaceae (4)	Quercus (1) Castanopsis (2) Cyclobalanopsis (1)
Flacourtiaceae (2)	Casearia (1) Homalium (1)
Gesneriaceae (1)	Conandron (1)
Gleicheniaceae (1)	Diplopterygium (1)
Gramineae (6)	Phragmites (1) Poa (1) Lophatherum (1)<br>Indocalamus (1) Eremochloa (1) Thysanolaena (1)
Grossulariaceae (1)	Itea (1)
Juglandaceae (2)	Juglans (1) Engelhardia (1)
Labiatae (3)	Meehania (1) Rabdosia (1) Lamium (1)
Lauraceae (13)	Cinnamomum (1) Cryptocarya (1) Lindera (2)<br>Litsea (2) Machilus (5) Neolitsea (2)
Leguminosae (5)	Vicia (2) Melilotus (1) Albizia (1) Sophora (1)
Liliaceae (7)	Trillium (1) Paris (1) Veratrum (1) Convallaria (1)<br>Lilium (1) Polygonatum (1) Allium (1)
Lindsaeaceae (2)	Lindsaea (1) Schizoloma (1)
Lygodiaceae (1)	Lygodium (1)
Magnoliaceae (1)	Schisandra (1)
Melastomataceae (4)	Melastoma (3) Memecylon (1)
Mimosaceae (5)	Albizia (1) Adenanthera (1) Pithecellobium (2) Leucaena (1)
Moraceae (10)	Artocarpus (1) Broussonetia (1) Ficus (8)
Myrsinaceae (4)	Maesa (1) Ardisia (2) Myrsine (1)
Myrtaceae (8)	Rhodomyrtus (1) Syringa (1) Syzygium (6)
Oleaceae (4)	Syringa (1) Fraxinus (1) Ligustrum (1) Jasminum (1)
Onagraceae (2)	Epilobium (1) Oenothera (1)
Osmundaceae (1)	Osmunda (1)
Oxalidaceae (1)	Averrhoa (1)
Palmae (2)	Calamus (1) Caryota (1)
Pandaceae (1)	Microdesmis (1)
Papaveraceae (1)	Hylomecon (1)
Phrymaceae (1)	Phryma (1)
Pinaceae (3)	Pinus (2) Picea (1)
Piperaceae (1)	Piper (1)
Pittosporaceae (1)	Pittosporum (1)
Podocarpaceae (1)	Podocarpus (1)
Polemoniaceae (1)	Polemonium (1)
Primulaceae (1)	Primula (1)
Pteridaceae (1)	Pteris (1)
Ranunculaceae (1)	Isopyrum (1) Aconitum (1) Aquilegia (1) Caltha (1)<br>Paeonia (1) Thalictrum (1)
Rhizophoraceae (1)	Carallia (1)
Rosaceae (15)	Amygdalus (1) Filipendula (1) Fragaria (1) Malus (1)<br>Padus (1) Photinia (1) Potentilla (1)<br>Pygeum (1) Rhaphiolepis (1) Rosa (1) Rubus (3) Spiraea (2)
Rubiaceae (10)	Aidia (1) Canthium (1) Cephalanthus (1) Diplospora (1)

	Morinda (1) Mussaenda (1) Psychotria (2) Rubia (1) Tarenna (1)
Rutaceae (3)	Phellodendron (1) Zanthoxylum (2)
Sabiaceae (1)	Meliosma (1)
Salicaceae (4)	Salix (2) Populus (2)
Santalaceae (1)	Dendrotrophe (1)
Sapindaceae (1)	Mischocarpus (1)
Sapotaceae (1)	Chrysophyllum (1)
Saxifragaceae (5)	Ribes (2) Philadelphus (1) Astilbe (1) Chrysosplenium (1) Ribes (1)
Selaginellaceae (1)	Selaginella (1)
Sterculiaceae (1)	Sterculia (1)
Symplocaceae (1)	Symplocos (1)
Thelypteridaceae (1)	Cyclosorus (1)
Theaceae (5)	Schima (1) Eurya (4)
Tiliaceae (2)	Tilia (2)
Ulmaceae (4)	Ulmus (1) Gironniera (1) Celtis (1) Trema (1)
Umbelliferae (6)	Bupleurum (1) Pimpinella (1) Anthriscus (1) Angelica (1) Heracleum (1) Sanicula (1)
Urticaceae (1)	Urtica (1)
Verbenaceae (2)	Clerodendrum (1) Vitex (1)
Vitaceae (1)	Cayratia (1)
Xanthophyllaceae(1)	Xanthophyllum (1)
Zingiberaceae (2)	Alpinia (2)

Table. S2 Relationships (R^2) between leaf hydraulic traits and anatomical traits.

Traits	Palisade thickness	Spongy thickness	Palisade / Spongy
Vein density	0.13	-	0.26
Stomatal length	0.13	-	-
Stomatal density	-	-	-
Maximum stomatal coductance	-	-	-

Data was drived from "The relationships between leaf economics and hydraulic traits of woody plants depend on water availability".

-, P > 0.05.

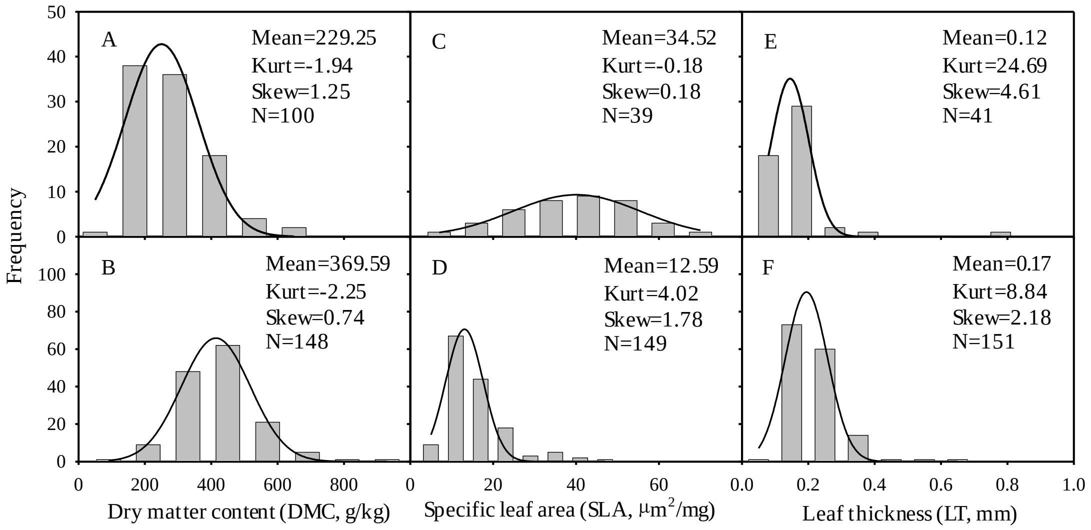

Fig.S1 Distribution of leaf morphological traits in temperate and subtropical forests.

A, C, E represent temperate forest; B, D, F represent subtropical forest.

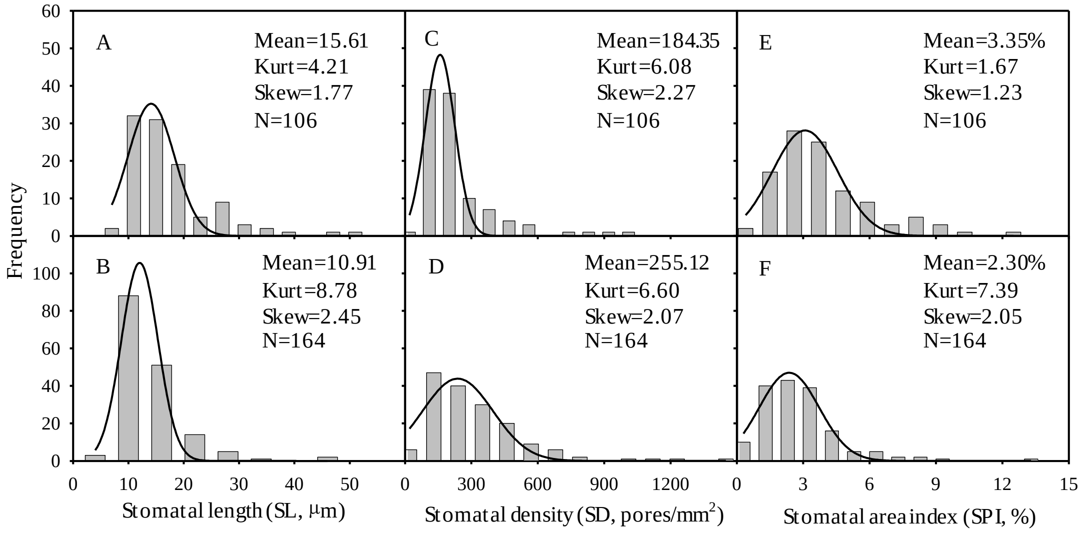

Fig.S2 Distribution of leaf stomatal traits in temperate and subtropical forests.

A, C, E represent temperate forest; B, D, F represent subtropical forest.

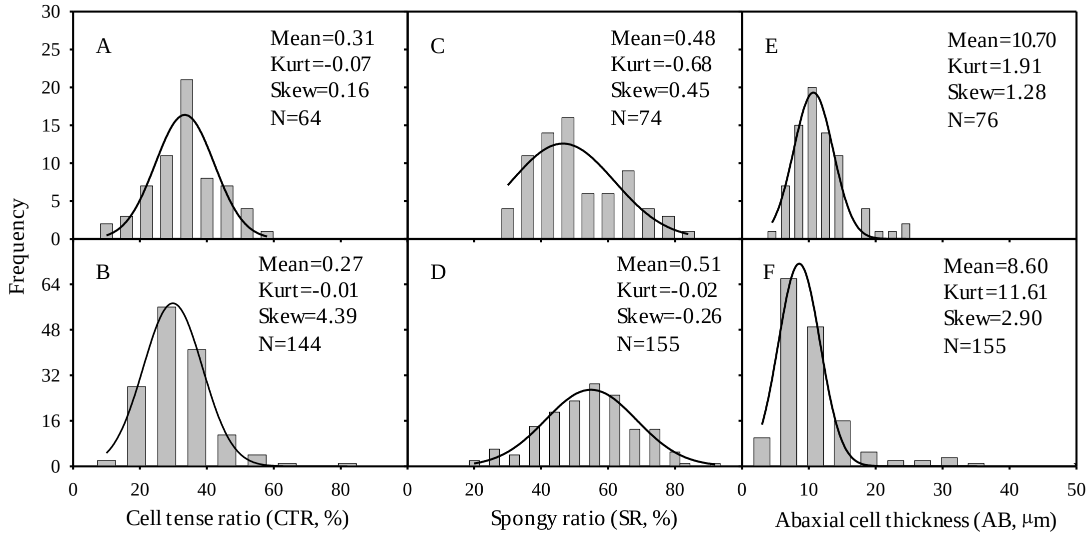

Fig.S3 Distribution of leaf anatomical traits in temperate and subtropical forests.

A, C, E represent temperate forest; B, D, F represent subtropical forest

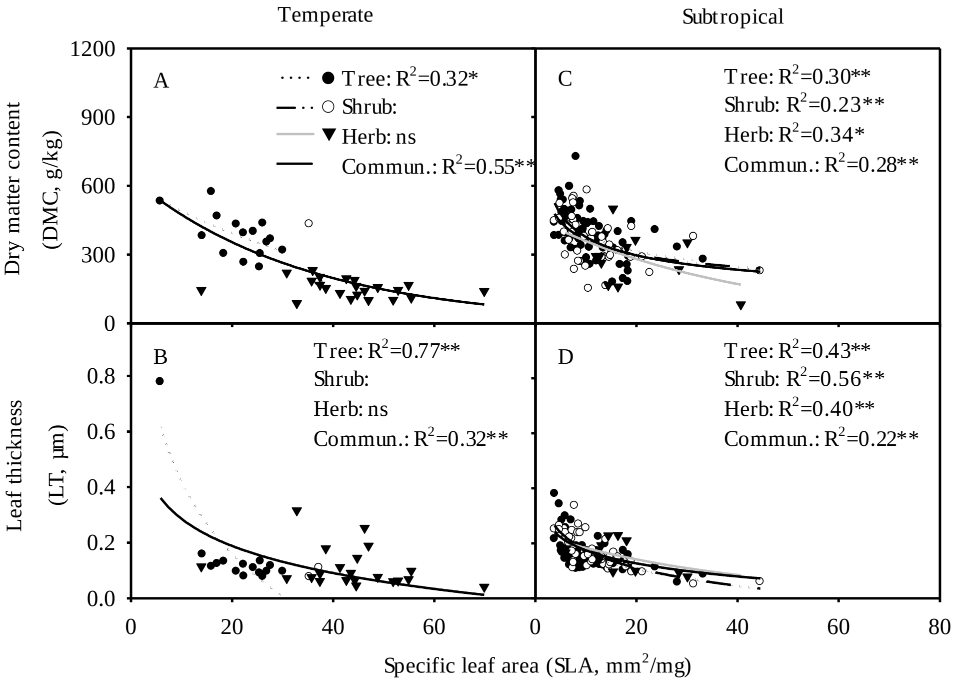

Fig. S4 Relationships between morphological traits at functional groups and communities.

ns, no significant difference; *, P < 0.05; **, P < 0.01. The regressions did not conducted because of few data of SLA for shrub observed in temperate forest (panels A and B).

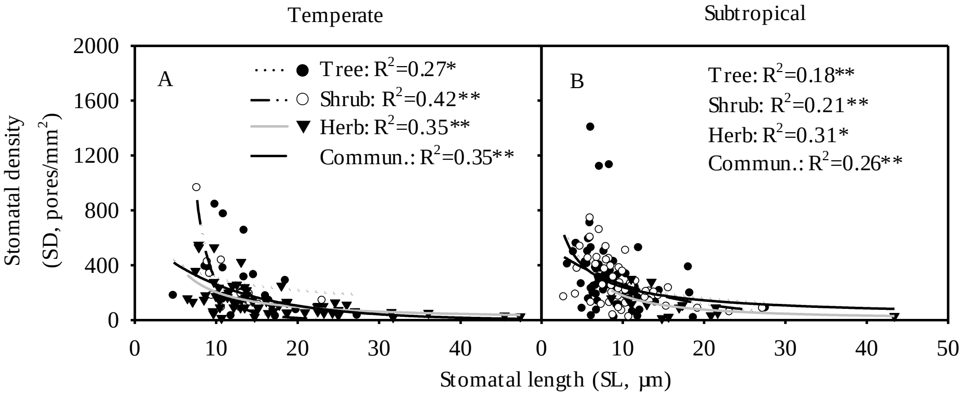

Fig. S5 Relationships between stomatal length (SL) and stomatal density(SD) at functional groups and communities. ns, no significant difference; *, $P < 0.05$; **, $P < 0.01$.

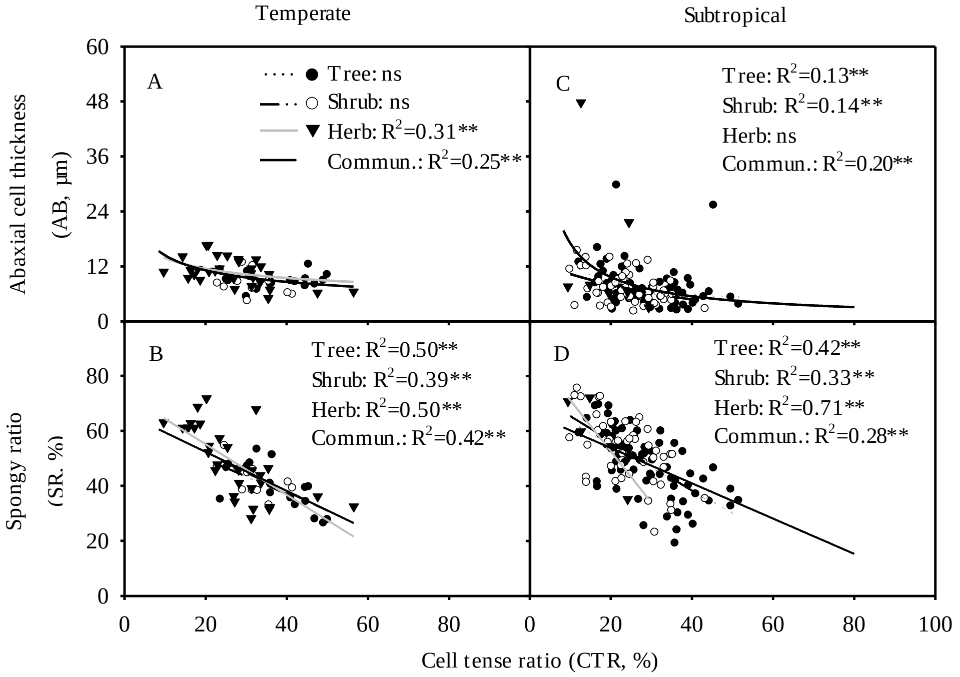

Fig.S6 Relationships among anatomical traits at functional groups and and communities.

ns, no significant difference; *, $P < 0.05$; **, $P < 0.01$.

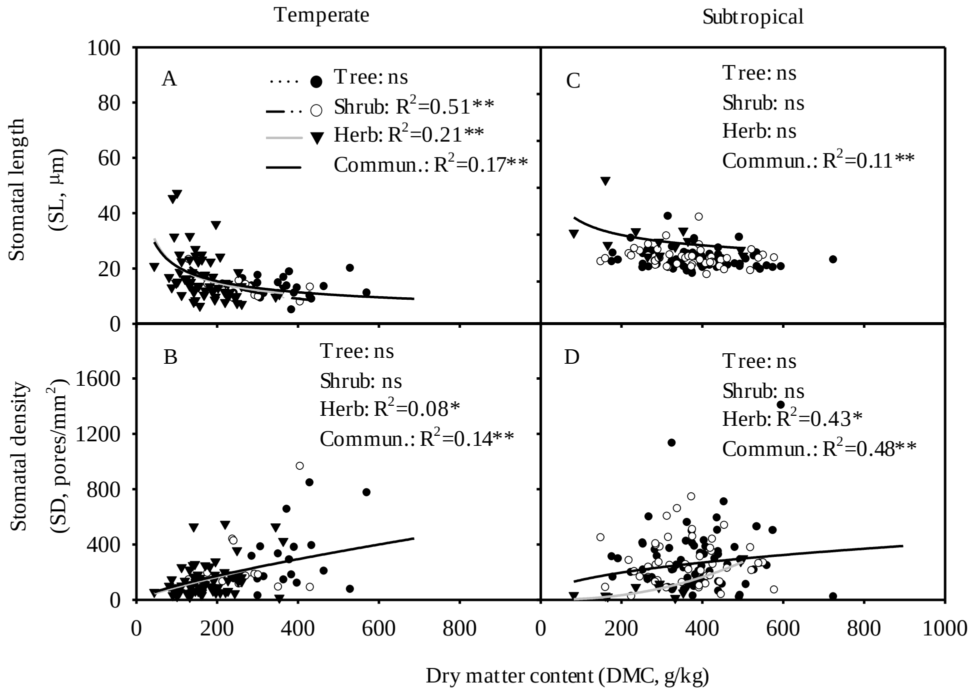

Fig. S7 Relationships between stomatal traits and dry matter content (DMC) at functional groups and communities.

ns, no significant difference; *, $P < 0.05$; **, $P < 0.01$.

.

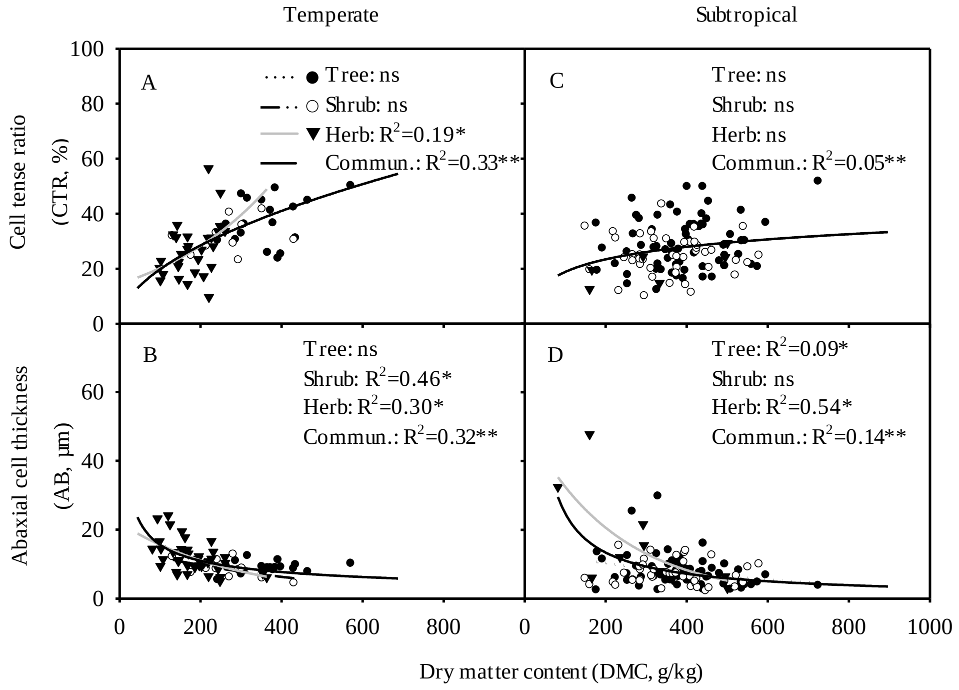

Fig.S8 Relationships between anatomical traits and dry matter content (DMC) at functional groups and communities.

ns, no significant difference; *, $P < 0.05$; **, $P < 0.01$.

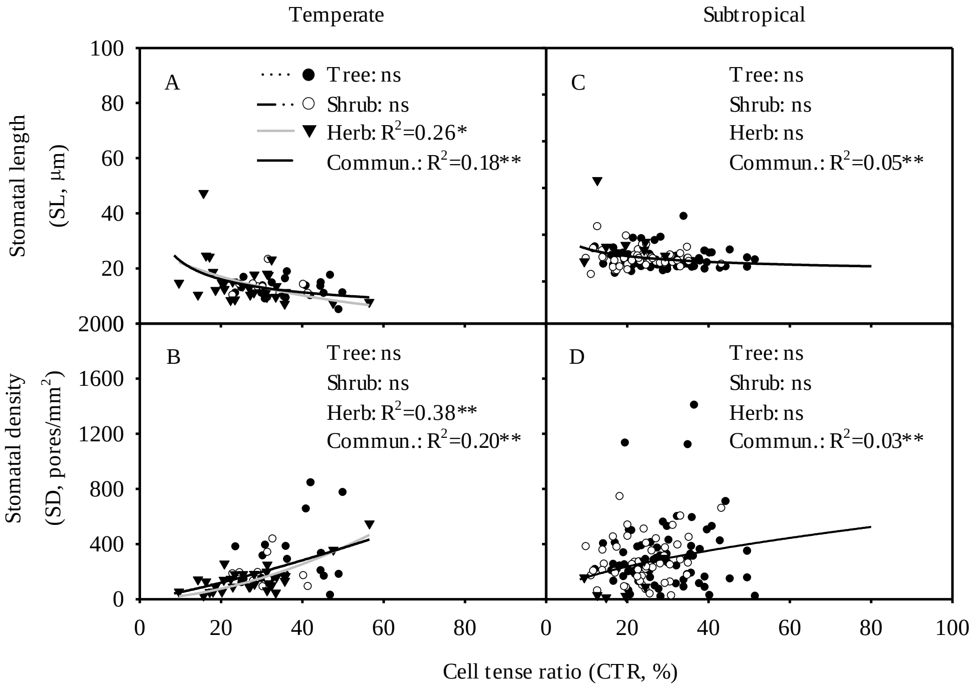

Fig. S9 Relationships between stomatal traits and cell tense ratio (CTR) at functional groups and communities. ns, no significant difference; *, $P < 0.05$; **, $P < 0.01$.

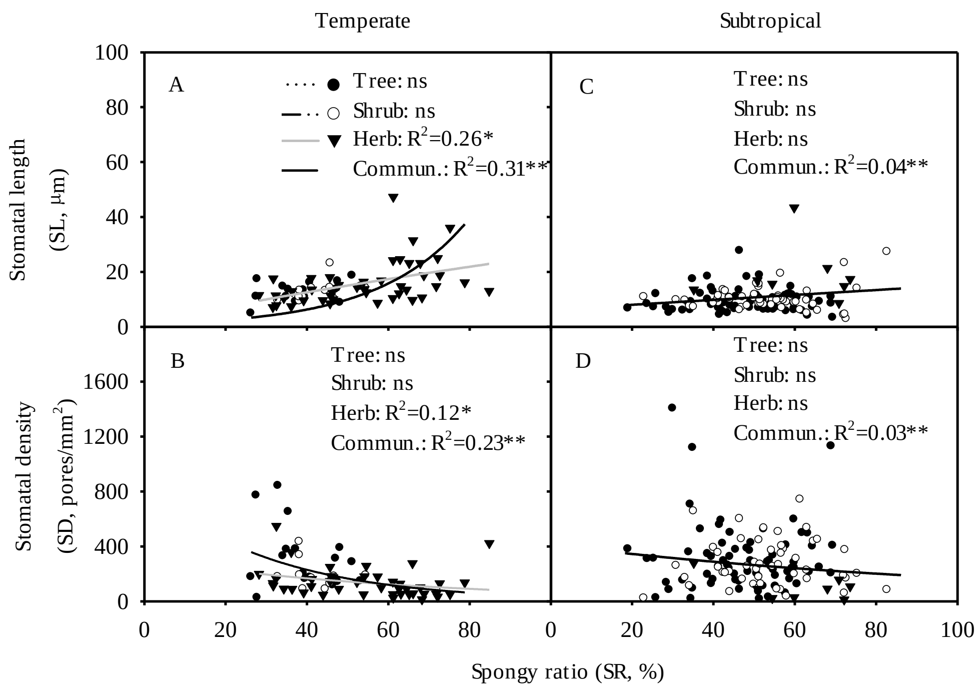

Fig. S10 Relationships between stomatal traits and spongy ratio (SR) at functional groups and communities. ns, no significant difference; *, $P < 0.05$; **, $P < 0.01$.

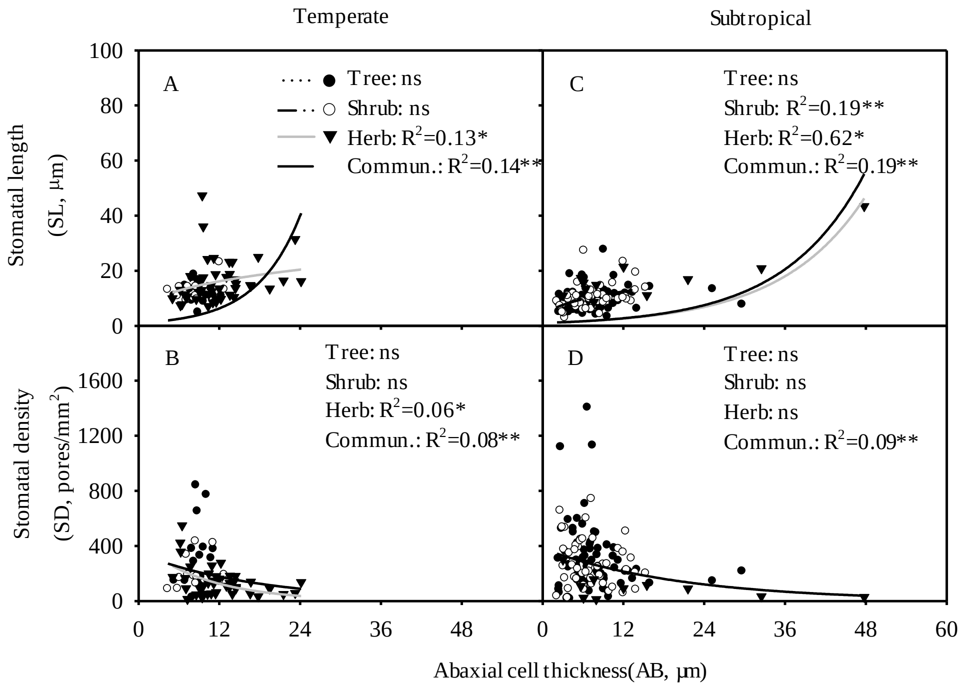

Fig.S11 Relationships between stomatal traits and abaxial cell thickness(AB) at functional groups and communities. ns, no significant difference; *, $P < 0.05$; **, $P < 0.01$.

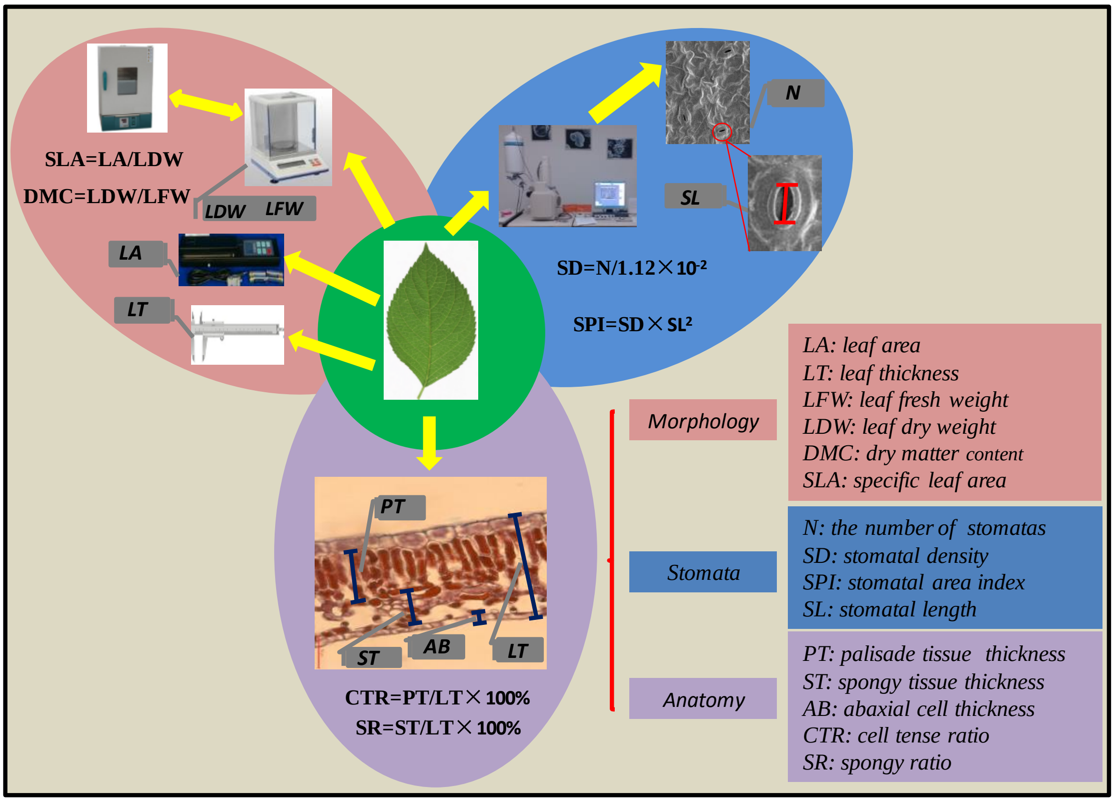

Fig. S12 Schematic diagram of the measured leaf traits in view of morphology, stomata, and anatomy.
